# Complete mitochondrial genome of a DHA-rich protist *Schizochytrium* sp. TIO1101

**DOI:** 10.1080/23802359.2016.1144090

**Published:** 2016-03-28

**Authors:** Zhaokai Wang, Sulin Lou, Fan Hu, Peng Wu, Longhe Yang, Huanqin Li, Lijuan He, Xiangzhi Lin

**Affiliations:** Engineering Research Center of Marine Biological Resource Comprehensive Utilization, Third Institute of Oceanography, State Oceanic Administration, Xiamen, China

**Keywords:** DHA, mitogenome, phylogeny, sequencing, Schizochytrium sp. TIO1101

## Abstract

*Schizochytrium* sp. TIO1101 is a crucial commercial alga used to produce docosahexaenoic acid (DHA), a long-chain polyunsaturated fatty acid that is beneficial for human health. In this study, we sequenced the mitochondrial genome (mitogenome) of *Schizochytrium* sp. TIO1101 for the first time using an Illumina HiSeq 2500 system (Illumina Inc., San Deigo, CA). The assembled mitogenome was 31 494 bp long with 33.92% GC content. The mitogenome contains 56 genes, including 33 protein-coding genes, 21 transfer RNA genes and two ribosomal RNA genes. Maximum-likelihood phylogenetic analysis of *Schizochytrium* sp. TIO1101 showed that it was most closely related to *Thraustochytrium aureum* among the examined species.

*Schizochytrium* sp. is a commercial alga used to produce docosahexaenoic acid (DHA), a long-chain polyunsaturated fatty acid, that is beneficial for human health (Metz et al. 2001; Burja et al. 2006; Fan et al. 2007). *Schizochytrium* sp. TIO1101, collected from Yundang Lake (Xiamen City, China), was isolated and characterized using pine pollen as bait and was deposited to China General Microbiological Culture Collection Center (CGMCC) with CGMCC no. 4603. The content of DHA in *Schizochytrium* sp. TIO1101 was reported to be 45% of the total fatty acids with an oil content up to 55% of the cell dry weight (Cheng et al. 2011). However, the metabolic mechanism for DHA synthesis in *Schizochytrium* sp. TIO1101 is unknown and until now only two gene sequences, those of 18S ribosomal RNA and malonyl-CoA acyl carrier protein transacylase (fabD), have been reported (Cheng et al. 2013). *Schizochytrium* sp. is considered to be a member of Stramenopiles, which are eukaryotes (Cavalier-Smith et al. 1994), whereas its reproductive style is characteristic of prokaryotes (Honda et al. 1998). Therefore, the evolutionary position of *Schizochytrium* sp. is still obscure. We sequenced the complete mitochondrial genome (mitogenome) of *Schizochytrium* sp. TIO1101, to obtain further insight into the phylogenetic position of *Schizochytrium* sp.

The complete mitogenome of *Schizochytrium* sp. TIO1101 was sequenced on an Illumina HiSeq 2500 platform (Illumina Inc., San Deigo, CA) and assembled *de novo* using SOAPdenovo2 (http://soap.genomics.org.cn). The assembled mitogenome sequence has been deposited in GenBank under accession number KU183024. To annotate the mitogenome, we used the online Dual Organellar GenoMe Annotator (Wyman et al. 2004) and ORF Finder programs (Cheng et al. 2013) with default conditions and the *Thraustochytrium aureum* mitogenome (GenBank accession no. AF288091.2) as a reference. We obtained a circular map 31 494 bp long with 33.92% GC content, consisting of 33 protein-coding genes (PCGs), 21 transfer RNA genes (tRNA) and two ribosomal RNA genes (rRNA) (Figure S1). The 33 PCGs comprised 15 ribosomal protein-coding genes (*rpl* and *rps*), 10 NADH dehydrogenase genes (*nad*), three ATP synthase genes (*atp*), three cytochrome c oxidase genes (*cox*), one cytochrome b gene (*cob*) and one sec-independent protein translocase component (*tatC*), sharing the same start codon (ATG) but different stop codons (TAA, TGA or TTA). The shortest PCG was *rps19* (165 bp) and the longest was *nad5* (1968 bp). All the tRNA genes were on the H-strand. The two rRNA genes encoded one large and one small subunit rRNA. The gene content and genome organization of the *Schizochytrium* sp. TIO1101 mitogenome are similar to that of the *T. aureum* mitogenome, indicating their genetic affinity.

The complete mitogenomes of 19 Stramenopiles species (including *Schizochytrium* sp. TIO1101) were used to construct a maximum-likelihood phylogenetic tree with nine Oomycetes species as the outgroup, using RaxML 8.1.5 software (Stamatakis 2006) (Figure 1). To build the phylogenetic tree, we used the translated amino acid sequences of 19 protein-coding genes that were conserved in the 19 examined species. The tree shows that *Schizochytrium* sp. TIO1101 was most closely related to *T. aureum* than to the other species.

**Figure 1.**
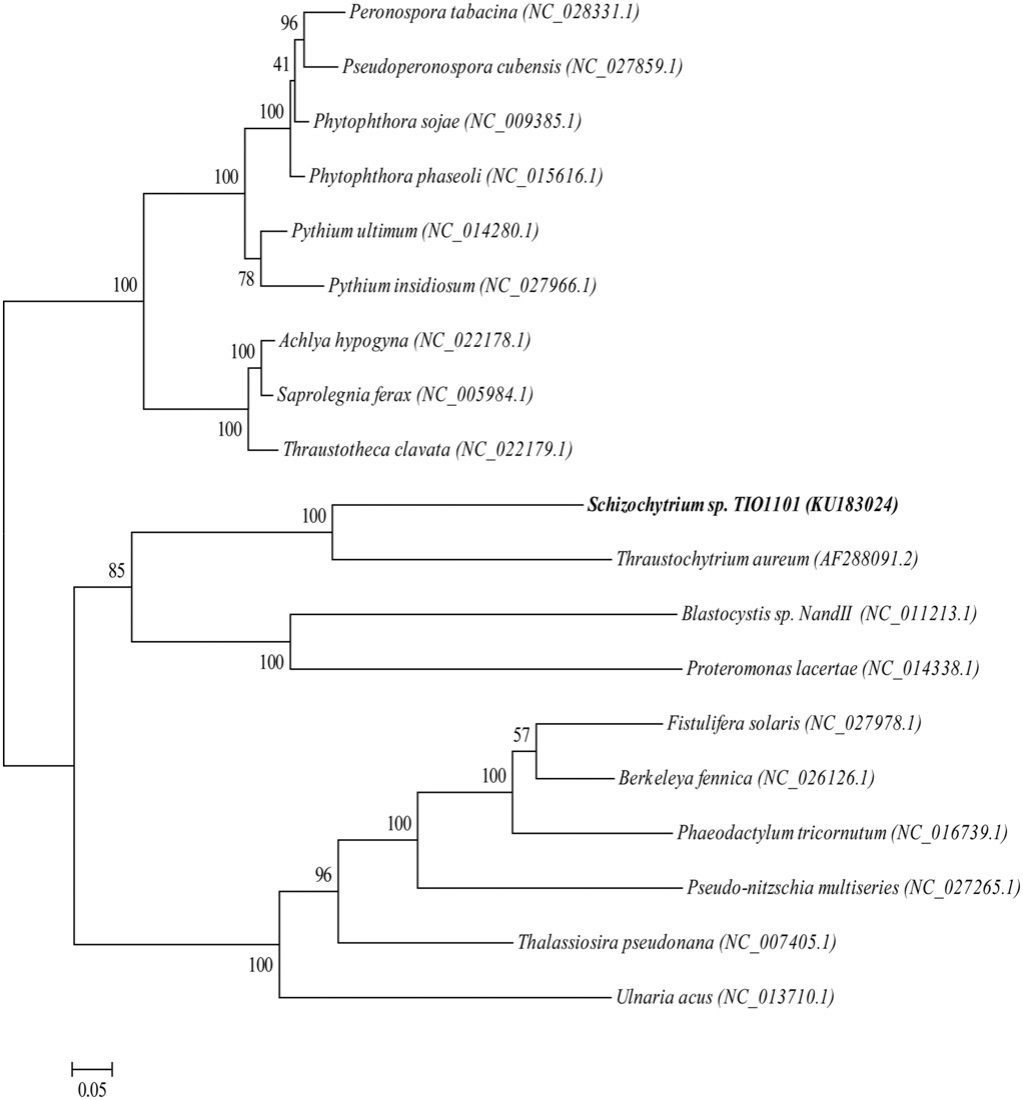
Phylogenetic analyses of *Schizochytrium* sp. TIO1101. A maximum-likelihood tree was constructed using amino acid of 19 mitochondrial protein-coding genes (*nad1, nad3, nad4, nad4L, nad5, nad6, nad7, nad9, atp6, atp9, rps12, rps13, rpl2, rpl14, rpl16, cox1, cox2, cox3* and *cob*) with 1000 replications of bootstrap re-sampling. GenBank accession numbers are provided after each species name. Organisms in bold text correspond to the species analyzed in this study.

## References

[CIT0001] Burja AM, Radianingtyas H, Windust A, Barrow CJ. 2006 Isolation and characterization of polyunsaturated fatty acid producing Thraustochytrium species: screening of strains and optimization of omega-3 production. Appl Microbiol Biotechnol. 72:1161–1169. 16625394 10.1007/s00253-006-0419-1

[CIT0002] Cavalier-Smith T, Allsopp MT, Chao EE. 1994 Thraustochytrids are chromists, not fungi: 18s rRNA signatures of heterokonta. Philos Trans R Soc Lond B Biol Sci. 346:387–397.

[CIT0003] Cheng JK, Zeng X, Ren GM, Liu ZH. 2013 CGAP: a new comprehensive platform for the comparative analysis of chloroplast genomes. BMC Bioinf. 14:95. 10.1186/1471-2105-14-95 PMC3636126 23496817

[CIT0004] Cheng RB, Ge YQ, Yang B, Zhong XM, Lin XZ, Huang Z. 2013 Cloning and functional analysis of putative malonyl-CoA: acylcarrier protein transacylase gene from the docosahexaenoicacid-producer *Schizochytrium* sp. TIO1101. World J Microbiol Biotech. 29:959–967. 10.1007/s11274-013-1253-0 23292648

[CIT0005] Cheng RB, Lin XZ, Wang ZK, Yang SJ, Rong H, Ma Y. 2011 Establishment of a transgene expression system for the marine microalga *Schizochytrium* by 18S rDNA-targeted homologous recombination. World J Microbiol Biotechnol. 7:737–741.

[CIT0006] Fan KW, Jiang Y, Faan YW, Chen F. 2007 Lipid characterization of mangrove thraustochytrid-Schizochytrium mangrovei. J Agric Food Chem. 55:2906–2910. 17381126 10.1021/jf070058y

[CIT0007] Honda D, Yokochi T, Nakahara T, Erata M, Higashihara T. 1998 Schizochytrium limacinum sp. nov., a new thraustochytrid from a mangrove area in the west Pacific Ocean. Mycol Res. 102: 439–448.

[CIT0008] Metz JG, Roessler P, Facciotti D, Levering C, Dittrich F, Lassner M, Valentine R, Lardizabal K, Domergue F, Yamada A, et al. (2001). Production of polyunsaturated fatty acids by polyketide synthases in both prokaryotes and eukaryotes. Science. 293:290–293. 11452122 10.1126/science.1059593

[CIT0009] Stamatakis A. 2006 RAxML-VI-HPC: maximum likelihood-based phylogenetic analyses with thousands of taxa and mixed models. Bioinformatics. 22:2688–2690. 16928733 10.1093/bioinformatics/btl446

[CIT0010] Wyman SK, Jansen RK, Boore JL. 2004 Automatic annotation of organellar genomes with DOGMA. Bioinformatics. 20:3252–3255. 15180927 10.1093/bioinformatics/bth352

